# Nutritional Therapy to Modulate Tryptophan Metabolism and Aryl Hydrocarbon-Receptor Signaling Activation in Human Diseases

**DOI:** 10.3390/nu12092846

**Published:** 2020-09-17

**Authors:** Mohammed Ghiboub, Charlotte M. Verburgt, Bruno Sovran, Marc A. Benninga, Wouter J. de Jonge, Johan E. Van Limbergen

**Affiliations:** 1 Department of Pediatric Gastroenterology and Nutrition, Amsterdam University Medical Centers, Emma Children’s Hospital, 1105 AZ Amsterdam, The Netherlands; m.ghiboub@amsterdamumc.nl (M.G.); c.m.verburgt@amsterdamumc.nl (C.M.V.); m.a.benninga@amsterdamumc.nl (M.A.B.); 2 Tytgat Institute for Liver and Intestinal Research, Amsterdam Gastroenterology and Metabolism, Academic Medical Center, University of Amsterdam, 1105 BK Amsterdam, The Netherlands; b.sovran@amsterdamumc.nl (B.S.); w.j.dejonge@amsterdamumc.nl (W.J.d.J.); 3 Department of Pediatric Surgery, Amsterdam University Medical Centers, 1105 AZ Amsterdam, The Netherlands; 4 Department of Surgery, University of Bonn, 53127 Bonn, Germany; 5 Department of Pediatrics, Dalhousie University, Halifax, NS B3K 6R8, Canada

**Keywords:** microbiota, tryptophan, AhR, nutritional therapy, human diseases

## Abstract

The aryl hydrocarbon receptor (AhR) is a nuclear protein which, upon association with certain endogenous and exogenous ligands, translocates into the nucleus, binds DNA and regulates gene expression. Tryptophan (Trp) metabolites are one of the most important endogenous AhR ligands. The intestinal microbiota is a critical player in human intestinal homeostasis. Many of its effects are mediated by an assembly of metabolites, including Trp metabolites. In the intestine, Trp is metabolized by three main routes, leading to kynurenine, serotonin, and indole derivative synthesis under the direct or indirect involvement of the microbiota. Disturbance in Trp metabolism and/or AhR activation is strongly associated with multiple gastrointestinal, neurological and metabolic disorders, suggesting Trp metabolites/AhR signaling modulation as an interesting therapeutic perspective. In this review, we describe the most recent advances concerning Trp metabolism and AhR signaling in human health and disease, with a focus on nutrition as a potential therapy to modulate Trp metabolites acting on AhR. A better understanding of the complex balance between these pathways in human health and disease will yield therapeutic opportunities.

## 1. Introduction

The mammalian intestine contains a complex symbiosis of host epithelial and immune cells, and luminal dietary antigens and microorganisms [1]. The intestine harbors a dense and diverse microbiota composed by commensal bacteria, fungi, archaea and virus communities [2]. These unique microbial communities have co-developed with the host to reach a symbiotic balance called homeostasis [3]. A loss of homeostasis, also called dysbiosis, has been described in several human diseases [2]. The intestinal microbiota plays a major role in many important physiological functions, such as metabolic, nutritional and immune homeostasis [4]. These effects are mediated by direct microbiota-host interactions and by microbial- and host-derived metabolites [4,5]. The intestinal microbiota serves as a virtual endocrine organ, producing and regulating multiple compounds that interact with host physiology and act to influence different functions at the local and distant levels [6]. Any disturbance in host-microbiota equilibrium can lead to a disease development. The host-microbiota interactions can be driven and controlled by a large range of metabolites [7]. For instance, (a) short-chain fatty acids (SCFAs), bacterial fermentation end products of dietary fibers, (b) bile acids, produced and recycled in the liver and (c) Tryptophan (Trp)-derivatives, are the most studied categories of metabolites implicated in host-microbiota cross talk [4,7].

Trp has been reported to play a key role in intestinal homeostasis [8,9]. Although the defect in Trp metabolism has been associated with multiple metabolic [9], gastrointestinal [10] and neurological disorders [11] and cancers [11], the pathological mechanism is poorly understood. Increasingly, the transcription factor aryl hydrocarbon-receptor (AhR) has emerged as a critical pathway for Trp metabolite ligands [12,13]. The AhR protein contains a DNA binding domain and a transcriptional activation binding domain, which indicates its role in transcription regulation [14,15]. Upon agonist binding (such as Trp-derived metabolites), AhR translocates to the nucleus, where it binds DNA and controls gene expressions in a ligand-specific, cell-type-specific and context-specific manner [16,17]. In this review, we report the most recent insights regarding the Trp metabolism/AhR axis in health and disease, with a focus on nutrition as a potential therapy to modulate adverse Trp metabolism acting on AhR.

## 2. Tryptophan Metabolism

### 2.1. Tryptophan Origin and Production

Trp is an essential aromatic amino acid that is acquired exclusively through dietary intake in humans (since Trp is not produced by animal cells) [18]. Common sources of dietary Trp are fish, poultry, cereals, and dairy foods [18]. The World Health Organization advises an intake of 4 mg of Trp/kg/day [4]. Trp is a precursor of several microbial and host metabolites [19], including several molecules such as serotonin, melatonin, nicotinamide and vitamin B3 [20].

### 2.2. Intestinal Tryptophan Metabolism Pathways

**Figure 1:**
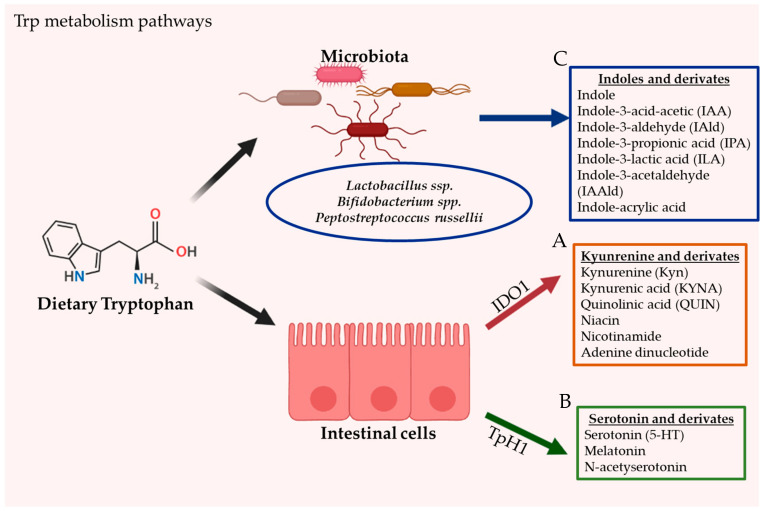
The gastrointestinal tract is the principal location of tryptophan (Trp) metabolism. In humans, Trp is acquired exclusively through dietary intake. Dietary Trp can be processed by three main metabolism routes: (**A**) the kynurenine pathway (KP), which mainly occurs in both immune and epithelial cells via IDO1, leading to several kynurenine metabolites (KYNs) including ligands for AhR (**B**) the serotonin production pathway, taking place in enterochromaffin cells (subtype of intestinal epithelial cells) and via TpH1, and finally (**C**) the direct conversion of Trp by the gut microbiota into several Indoles and derivates, including ligands of the AhR.

The intestine is a prime location of Trp metabolism. Three main metabolic pathways can process dietary Trp: (i) the kynurenine pathway (KP) via indoleamine 2,3-dioxygenase (IDO)1, mainly occurring in both immune and epithelial cells [21], (ii) the serotonin (5-hydroxytryptamine [5-HT]) pathway via Trp hydroxylase 1 (TpH1) in enterochromaffin cells [22], and finally (iii) the direct conversion of Trp by the intestinal microbiota into several molecules, including ligands of the AhR [23]. Trp metabolism pathways are illustrated in Figure 1.

#### 2.2.1. Kynurenine Pathway (KP)

Intestinal Trp metabolism through the KP is initially mediated by the enzyme Indoleamine 2,3-dioxygenase (IDO)1 and leads to the production of kynurenine pathway metabolites (KYNs); such as kynurenine (Kyn), quinolinic acid (QUIN), niacin, nicotinamide adenine dinucleotide and kynurenic acid (KYNA) [24,25]. Trp 2,3-dioxygenase (TDO) and IDO2 also participate in Trp metabolism to form Kyn. However, these enzymes are not expressed in the gut. Over 95% of Trp has been reported to be metabolized via the KP in mammals [26,27].

The gut microbiota has a critical role in stimulating IDO1 activity [28]. This was demonstrated in germ-free and antibiotic treated mice [28]. Depletion of Trp by IDO1, IDO2 and TDO can have fundamental consequences on cellular function and survival [24]. Several intestinal bacteria produce homologous enzymes and are thus also capable of Kyn synthesis and downstream neurotoxic metabolites such as 3-hydroxyanthranilic acid [29,30]. It has been reported that KYNs are implicated in host biological processes such as cell differentiation, neurotransmission, inflammation, immune response [4], as well as its affinity to AhR [12,13].

#### 2.2.2. Serotonin Pathway

The neurotransmitter serotonin, also known as 5-HT, is a monoamine neurotransmitter that is mostly produced by a specialized subtype of intestinal epithelial cells called enterochromaffin in the gastrointestinal tract [31,32]. Trp is initially processed by TpH1 to 5-HTP metabolite, then further metabolized to serotonin. Serotonin produced in the enterochromaffin cells is eventually released to the blood. Peripheral serotonin participates in several intestinal functions and is involved in several human physiological functions by activating the serotonin receptors [33]. Serotonin is an important gastrointestinal signaling substance that transmits signals from the intestine to the neuronal network and influences intestinal motility, secretion, vasodilatation, and the absorption of nutrients. The intestinal microbiota is a key player for the production of intestinal serotonin [22]. Germ-free mice have demonstrated impaired production of serotonin in the colon and low concentration of circulating serotonin. While the mechanism modulating the production of serotonin by the intestinal microbiota remains poorly understood, SCFAs have been suggested to have a role in the stimulation of TpH1 expression [34]. In addition, some secondary bile acids, such as deoxycholate synthesized by microbial biotransformation of cholate, can also stimulate serotonin biosynthesis [22]. Serotonin can also be produced in the brain through TpH2 enzyme in serotonergic neurons, where it regulates major physiological properties such as mood, appetite, and sleep [31,32].

#### 2.2.3. Direct Trp Metabolism by Microorganisms

Through its metabolic activity, the intestinal microbiota orchestrates the direct Trp degradation into indole derivatives, including indole-3-acid-acetic (IAA), indole-3-aldehyde (IAId), indole-3-propionic acid (IPA), indole-3-lactic acid (ILA), indole-3-acetaldehyde (IAAId) and indole-acrylic acid [35,36,37]. Indole is an aromatic organic compound produced by a variety of bacteria [38]. These ligands are able to affect bacterial physiology and to modulate antibiotic resistance, sporulation, and biofilm formation [38]. Although the role of the microbiota in this process is critical, only a few commensal species such as *Bifidobacterium* spp. [37], *Peptostreptococcus russellii* [39] and *Lactobacillus spp.* [23,40] have been well characterized to contribute to Trp metabolism. In these species, the oxidative/reductive pathways lead to the production of IAA and IPA, two Trp metabolites known to improve the intestinal barrier and boost host immunity [40,41,42]. The metabolism of Trp into AhR ligands by *Lactobacilli* has been shown to control intestinal colonization by pathogenic *Candida albicans* via AhR dependent IL22 production [23]. *Lactobacillus reuteri* and *Lactobacillus johnsonii* have been identified to produce the indole derivative IAId, generated through the indole pyruvate pathway and catalyzed by the enzyme aromatic amino acid aminotransferase [23].

Tryptophanase, an enzyme expressed in *E. coli* and *lactobacilli*, was described to be involved in Trp conversion into indole [36]. Indole is transported in and out of the bacteria by passive diffusion through the membrane or actively with AcrEF-TolC and Mtr transporters [43,44,45]. Exogenous diet-derived indole molecules, such as Trp and glucobrassicin, are a major source of endogenous AhR ligand precursors [36,46,47].

## 3. Origins of AhR Ligands

The AhR is capable of binding diverse groups of ligands that display varying levels of affinity and reactivity [48]. AhR was originally described as a sensor of xenobiotic chemicals. However, it has more recently been discovered that AhR can also be activated or deactivated by numerous endogenous ligands [9,49].

AhR ligands can be classified as either from exogenous or (pseudo-) endogenous origin [9]. Exogenous ligands can be xenobiotics such as aromatic (aryl) hydrocarbons, which define the name of the receptor [50]. These can be environmental pollutants, like polycyclic aromatic hydrocarbon (PAH) and halogenated aromatic hydrocarbon (HAH) [50]. The majority of high-affinity AhR ligands are exogenous synthetic chemicals [51]. In fact, the AhR was first known due to its implications in clinical manifestations following 2,3,7,8-tetrachlorodibenzo- p-dioxin (TCDD), a type of HAH, causing a range of toxic effects including immune suppression, tumor promotion and altered cell differentiation [52,53]. Exogenous ligands can also originate from the diet, like Trp, flavonoids, curcumin and carotenoids [36].

Endogenous ligands are predominantly dietary-derived ligands, i.e., pseudo-endogenous ligands, that are metabolized either by UV light exposure, host cells or by bacteria in the gut [51,54]. The major dietary-derived precursors for AhR ligands originate from the essential amino acid Trp [55]. Trp can undergo a range of conversions following light exposure, host enzymatic activity or modulation by microbiota [36]. One of Trp’s most significant photoproducts is 6-formylindolo [3,2-b] carbazole (FICZ), which is a very potent AhR agonist and is therefore used as a positive control in numerous experimental models investigating AhR [56]. Host enzyme-catalyzed reactions are regulated by IDO1, leading to AhR ligand metabolites such as Kyn, quinolinic acid, nicotinamide, niacin and KYNA, or by 5′HT, leading to serotonin, melatonin or n-acetylserotonin [57]. Microbiota species like *Lactobacillus* ssp., *Bifidobacterium* ssp. and *Peptrostreptococcus Russellii* are able to form various Trp derivatives (AhR ligands) like Indole, IIAA, IAId, IPA, ILA, IAAId and indole-acrylic acid [35,36,37] (Figure 1). However, many potential AhR-ligand-forming microbiota species likely remain to be identified. Glucobrassicin is derived from IAAId forms into indole-3-carbinol (I3C) and indole-3-acetonitrile (I3AC) by enzymatic cleavage, which in turn can form a potent AhR agonist called indolo [3,2-b] carbazole (ICZ) in the presence of gastric acid [46]. It is important to highlight that AhR ligands are known to have varying species- and tissue specific effects. AhR ligands can either agonize or antagonize the receptor depending on the administered concentration. In the presence of numerous ligands, they can even become competitive agonists or antagonists of each other [9].

## 4. AhR Functional Domains and Signaling

AhR is expressed ubiquitously by cells throughout the body [58,59]. It is present in the cytoplasm of cells in its inactive form and exists as a multi-protein complex with two chaperone proteins: heat shock protein (Hsp) 90 and the co-chaperone p23 [60].

The AhR protein contains several highly conserved domains with distinct functions that are critical for its activity [61]. The first is the basic-region, involved in the binding of the transcription factor to DNA [61]. The second is the helix-loop-helix region, which facilitates interaction with ligands [61]. AhR contains two PAS domains, PAS-A and PAS-B which are highly homologous to the aryl hydrocarbon receptor nuclear translocator (ARNT) [61]. The AhR ligand binding site is located in the PAS-B domain and is composed of several conserved residues critical for ligand binding [61]. Finally, a glutamine-rich domain located in the C-terminal region of the protein is involved in co-activation and transactivation [61]. Upon AhR activation (following association with ligand), AhR ligands are processed and inactivated by cytochrome p450 family proteins (such as CYP1A1). The latter is a direct AhR transcription factor constituting a feedback loop for AhR signalling [62]. At activation, the AhR complex changes conformation and translocates into the nucleus, where AhR, and its ligand detach from the rest of the chaperone proteins [63].

AhR has been characterized as a cytoplasmic receptor able to bind to a variety of exogenous and endogenous ligands [51]. Despite its reactivity to different ligands, the AhR pathway is thoroughly controlled by three checkpoints that regulate AhR activation: (a) degradation of AhR in the proteasome, (b) metabolism of ligands by CYP1A1, and (c) disruption of the AhR:Arnt complex by AhRR [64].

In the intestine, the AhR pathway is a key actor of homeostasis. Its activation by specific agonists is crucial for initiating immune response and modulating epithelial renewal and barrier integrity [28,65,66]. It also acts on many immune cell types, such as intraepithelial lymphocytes, Th17 cells, innate lymphoid cells, macrophages, dendritic cells (DCs), and neutrophils [28,65,66]. Trp metabolism and AhR signaling have been implicated in several neurological, metabolic and gastrointestinal diseases [4].

## 5. Tryptophan Metabolism Pathways and AhR in Disease

**Figure 2:**
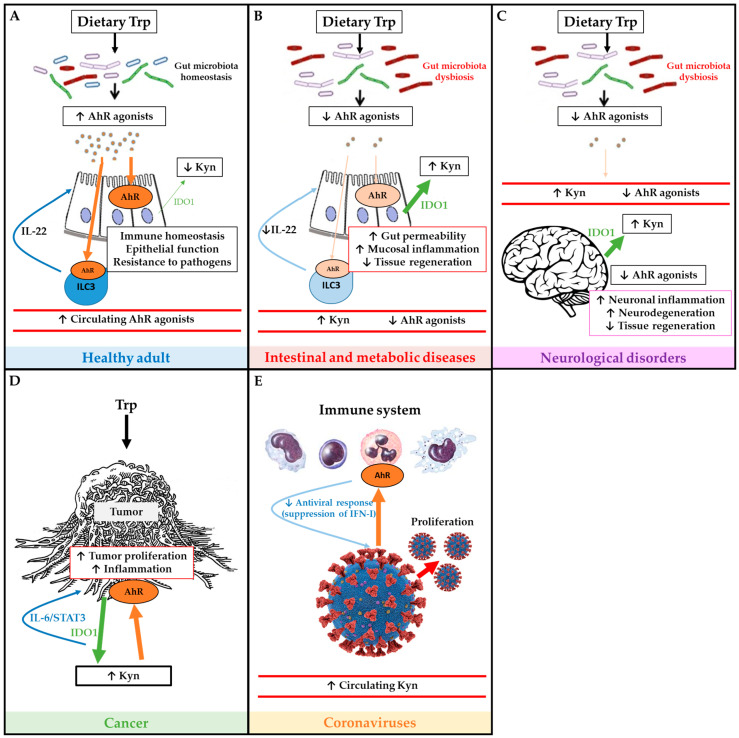
Schematic overview of tryptophan (Trp) metabolism and AhR pathway in health and disease. Panel (**A**) depicts the Trp metabolism and AhR pathway in a healthy adult, and its roles on immune and epithelial homeostasis. Panel (**B**) illustrates how IBD and metabolic diseases compromise the AhR pathway which affects intestinal permeability, inflammation and tissue regeneration. Panel (**C**) describes how neurological disorders affect Trp metabolism and AhR pathway in order to worsen symptoms of neurodegeneration. Panel (**D**) illustrates how tumors use Trp and activate AhR in order to proliferate. Panel (**E**) depicts immune AhR activation by coronaviruses in order to inhibit antiviral response and promote proliferation. Green arrows represent IDO1 production, large green arrows represent high production and the narrow arrows represent low production. Brown arrows represent AhR activation, large brown arrows represent high activation of AhR, and the narrow arrows represent low activation of AhR.

Some of the main effects of Trp metabolism and AhR pathway in health and disease are illustrated in Figure 2.

### 5.1. Metabolic Diseases

Obesity and metabolic syndrome have been associated with a dysregulation of tryptophan metabolism [9]. In metabolic syndrome, a decreased ability of the microbiota to produce AhR ligands has been described in mice and humans, leading to defective intestinal barrier integrity and reduced GLP-1 secretion [67]. In patients suffering from metabolic syndrome, an overactivation of IDO1 has been reported with increased Kyn levels in serum and increased Kyn/Trp ratio. These have been correlated with obesity (BMI), metabolic syndrome and blood triglycerides [68]. IDO deletion or inhibition has been shown to improve insulin sensitivity, preserve the intestinal barrier, decrease endotoxemia and chronic inflammation, and regulate lipid metabolism in liver and adipose tissues [28].

In mice, supplementation of diet with AhR agonists or a *Lactobacillus* strain naturally producing AhR ligands improved metabolic impairments [67]. Furthermore, the AhR agonist indigo has been described to protect against obesity-related insulin resistance through modulation of intestinal and metabolic tissue immunity [69]. Lin et al., suggest that the AhR ligand indigo has therapeutic potential to modulate inflammatory signals in obesity. Moreover, AhR activation is known to decrease serum glucose and triglyceride concentrations, described highly increased in patients suffering from obesity and metabolic syndrome [69]. AhR deficiency has been linked with dyslipidemia and dysbalanced glucose homeostasis, two parameters known to contribute to the development of type 2 diabetes in humans [70]. Thus, AhR ligands described to have the capacity to decrease the symptoms of metabolic diseases represent an important avenue of new therapy development.

### 5.2. Inflammatory Diseases

The interaction between AhR and its ligands that mainly originate from the diet or are metabolized by microbiota, is crucial in maintaining intestinal homeostasis [54]. Several gastrointestinal diseases, including Inflammatory Bowel Disease (IBD), are characterized by severe dysbiosis [10] and altered nutrient availability in the gut [71], which ultimately leads to less availability of AhR ligands like Trp metabolites. Patients with IBD have been shown to have less serum Trp levels compared to healthy controls [72]. Similarly, an increase in clinical disease activity (and need for intestinal resection) has been linked to low serum Trp levels [73]. In ulcerative colitis (UC), the KYNA/Trp ratio correlated with endoscopic and histologic disease activity [73].

As Trp originates solely from the diet [20], decreased levels can be due to altered intake associated with specific dietary patterns or reduced bio-availability due to altered uptake. In addition, altered AhR and related enzyme expression also seem to play a role in GI disease. Mononuclear cells of intestinal tissue and lamina propria from patients with IBD have been shown to express significantly less AhR compared to healthy controls [20,74]. Furthermore, IDO1 expression is significantly increased in patients with active disease. This was confirmed by the observation that IDO1 expression significantly correlated with endoscopic disease activity in a large UC cohort taking into account various other relevant UC parameters [73]. The strong changes in AhR expression in rodent/human disease models compared to healthy controls, suggest a mechanistic role of AhR in disease development [75,76].

Studies have shown an increased susceptibility to experimental colitis in AhR-deficient mice compared to control mice, suggesting that a deficiency in AhR ligands, followed by a lack of AhR activation in the intestine, may worsen inflammation [75,76]. Induction of IL-22 expression is one of the main outcomes of the activation of AhR signaling [77]. IL-22 has been suggested as a critical anti-inflammatory mediator in colitis [78,79]. IL22 knockout mice failed to attenuate the inflammatory reaction. Administration of FICZ, a potent AhR agonist, has been shown to reduce IFN-γ production and upregulate IL-22 in mice [72]. CARD9 is a susceptibility gene for IBD development and functions in the immune response against microorganisms and promotes IL-22 induction [80,81]. It has been described that the intestinal microbiota from CARD9 deficient mice failed to metabolize Trp into AhR ligands, increasing susceptibility to colitis and decreasing epithelial cell proliferation and increased apoptosis [40]. Transfer of CARD9-deficient microbiota into wild type or germ free mice, increased their susceptibility to colitis, with IL-22 being the most highly downregulated gene compared to controls [40]. The observation that mice lacking IL-22 induction ability are more prone to develop colitis, illustrates the central role for IL-22 in regulating the development of colitis. Another study suggested that AhR reduces inflammation in experimental colitis via the MK2/p-MK2/TTP pathway [76]. The MK2/p-MK2 pathway regulates stability, expression and function of tristetrapolin (TTP), an RNA binding protein of the TIS11 family [82]. TTP promotes degradation of numerous inflammatory factors upon binding to its target mRNA [83]. In DSS-colitis mice receiving FICZ treatment, TTP expression was increased along with p-MK2 downregulation [76]. However, MK2 expression was not altered. Subsequently, when *AhR* gene expression was knocked-down, FICZ was uncapable to downregulate p-MK2 expression [76]. These findings suggest that FICZ upregulates the expression of TTP by downregulating p-MK2 expression [76]. Together, these findings in rodent models suggest that disruption of the Trp metabolites/AhR signaling axis is a key mechanism in colitis development. The exact mechanism remains to be confirmed and supported in humans.

Increased Trp degradation (and increased level of Kyn) has also been associated with rheumatoid arthritis and systemic lupus erythematosus [84]. AhR is a key regulator of T cell subset differentiation, including Th17 and Treg cells, as well as several functions of DCs and macrophages [85]. Furthermore, AhR deficiency in T cells suppresses the development of collagen-induced arthritis in mice [86].

### 5.3. Neurological Disorders

The dysregulation of Trp metabolism has been associated with a range of neurodegenerative, neurological, neurogenesis and psychiatric disorders [11,87]. While Trp is an important precursor for production of the neurotransmitter serotonin, several catabolites along the Kyn axis are also neuroactive [88]. The KP for example, has demonstrated an important role in regulating the synthesis of both neuroprotective (kynurenic and picolinic acid, and the cofactor NAD+) and neurotoxic metabolites (QUIN and 3-hydroxykynurenine (3-HK) [88]. KYNA/Kyn, KYNA/QUIN and KYNA/3-HK are ratios used to estimate the balance between the neuroprotective and the neurotoxic metabolites, which reflects the neurotoxicity and neurodegeneration [89]. AhR is expressed in several types of neural cells, such as neurons, astrocytes and microglial cells [90]. A decrease in circulating AhR agonist levels, originating from gut microbiota, has been described in multiple central nervous system (CNS) diseases [91].

The function of AhR has been mainly studied using the organic pollutant TCDD but also using a non-genotoxic xenobiotic [90]. KYNs (AhR endogenous ligands) can pass through the blood-brain barrier, supplying additional peripheral KYNs to the brain, and the KP is highly activated in astrocytes and microglia in the brain [92]. This indicates that AhR activation by KYNs may play a critical role in numerous physiological and pathological processes influencing neurogenesis [93,94], cell proliferation [95,96], differentiation [94], and survival [90] in the nervous system.

Recent studies have shown that AhR can reduce pro-inflammatory cytokine expression in astrocytes [97] and microglia [97], which play a role in plasticity [98] and influence the development of multiple sclerosis (MS) [99], Alzheimer’s disease [100] and epilepsy [101]. Microbial catabolites of dietary Trp activate AhR signaling in astrocytes and suppress CNS inflammation in murine experimental autoimmune model of MS, whereas MS patients have lower circulating levels of AhR agonists [102]. AhR signaling activation during induction of experimental autoimmune encephalomyelitis (EAE) causes accelerated onset and increased pathology in wild-type mice, but not *AhR*-deficient mice [103]. It has been demonstrated that AhR modulates the function of dendritic cells and T cells to reduce the severity of the EAE [104].

In Huntington’s disease, neuroprotective KYNA levels are decreased in several regions of the brain [105]. It was demonstrated that in AhR deficient mice, KYNA levels are increased, conferring a neuroprotective effect against excitotoxic insult and oxidative stress [106]. Moreover, germ-free mice exhibit a deficiency in AhR agonists and show increased susceptibility to chronic stress and anxiety and depression-like behavior [107]. Furthermore, in a study using LPS-induced depression model, Kyn was associated with increased systemic inflammation-induced monocyte trafficking, mediating neuroimmune dysregulation and enhanced depression-like behavior [108]. Contrary, pharmacological inhibition of AhR and circulatory monocyte clearance decreased levels of LPS and Kyn and reduced the depressive symptoms in mice [108], indicating that Kyn and AhR are critical for immunoregulation and depression.

Trp-AhR signaling was also suggested to have some effects on the circadian rhythm [109]. When a rat CNS cell line is treated with Trp metabolites, an alteration in the expression of circadian genes (including *Per1*) was observed, suggesting that AhR signaling activation by exogenous or endogenous ligands may have an impact on the circadian rhythm [109].

Xenobiotic AhR ligands may exert their toxicities in CNS by competitive binding of AhR with endogenous ligands, including Trp metabolites such as Kyn and indoles [109]. Studies have demonstrated that different AhR ligands do not induce similar transcriptional responses in the brain; according to the nature of its ligand, the AhR may bind different responsive elements which vary in one or two bases [109].

### 5.4. Cancer

Trp metabolism has been strongly linked to cancer pathogenesis [11,110,111]. In physiological conditions and as a defense mechanism, local inflammation depletes Trp, limiting growth of microbes and proliferating malignant cells [110,111]. In tumor conditions, the cells develop countermeasures via increasing the Trp degradation and accumulation of Trp metabolites, leading them to suppress the tumor immune response and escape that defense mechanism [110,111]. An overactivation and overexpression of Trp-degrading enzymes IDO1 was observed in different types of cancer [112,113], promoting tumor progression by reducing antitumor immune responses and increasing the malignant properties of cancer cells [11,112]. The stimulation of IDO1 accelerates the degradation of Trp into KYNs such as Kyn, impacting Trp availability for immune cells, which can modulate anti-tumor immune responses [114]. Circulating Trp levels were decreased in patients with T cell leukemia, colorectal cancer [115] and other cancer types [116,117]. Enhanced Trp breakdown observed in cancer patients is often reflected by an increase in peripheral Kyn concentration and poor clinical outcome [111,118]. Furthermore, Niranjan et al. have demonstrated elevated Trp-metabolizing enzymes and KYNs in colon cancer cell lines, as well in human colon cancer tissues adenocarcinoma [119]. The increase in KYNs is suggested to play an important role in the pathophysiology of tumor immune tolerance via activation of AhR signaling [119].

Recent studies suggest that AhR activation enhances the initiation, promotion, progression, invasion, and metastasis of cancer cells [120]. Increased AhR expression and nuclear translocation was detected in invasive and malignant tumor cell lines [121,122]. Studies in a human adenocarcinoma cell line showed that DNA binding with the AhR was required for the cell cycle [123]. This interaction with an AhR agonist could transform the AhR into its DNA-binding form in order to stimulate the growth of cancer cells [123]. The interaction between AhR and Kyn can be inhibited with CH223191 in order to reduce the proliferation of colon cancer cells and increase preferential death of colon cancer cells [119]. This suggests that inhibition of Trp breakdown or KYNs/AhR signaling might be a promising therapeutic target in cancer to modulate the immunosuppressive tumor microenvironment. Jing et al. have shown that combining carboxyamidotriazole (anti-cancer molecule) with IDO1-Kyn-AhR pathway inhibitors profoundly enhanced cancer immunotherapy in primary tumor cells isolated from tumor-bearing mice [124].

KYNs/AhR signaling has remarkable effects on the immune response during cancer progression. KYNs binding to AhR have been shown to alter the proliferation and function of several immune effectors, including CD8+ T cells, and provide tumor cells with a means to elude anticancer immunosurveillance [125]. Kyn activates the AhR-ARNT associated transcription of IL-6 in human cancer cell lines, provoking autocrine activation of IDO1 via STAT3 [126,127]. This AhR-IL-6-STAT3 loop is correlated with a weak prognosis in lung cancer, favoring the idea that IDO-mediated immunosuppression enables the immune evade of tumor cells [126,127].

### 5.5. Coronaviruses

The ligand activated AhR controls several aspects of the immune response [65]. In addition to the capability of AhR to be activated by metabolites, certain viral infections can activate AhR signaling, which in turns dampens the immune response against viruses, e.g., the suppression of IFN-I [102]. AhR activation was shown to be associated with certain type of Zika and dengue viruses [128,129]. AhR antagonists are capable of activating anti-viral immunity, diminishing the viral load and improving Zika virus-induced pathology in vivo [128]. Transcriptional analysis of the in vivo and in vitro response to infection by multiple coronaviruses has detected an improved expression of the AhR transcriptional targets *CYP1A1* and *CYP1B1* [129]. Interestingly, AhR has been shown to be highly activated during coronavirus infections, including MERS-CoV [130], SARS-CoV-1 [131] and SARS-CoV-2 (responsible for the new outbreak COVID-19) [131]. AhR signaling is implicating in the response of lung basal cells, which give rise to stem cells involved in lung repair, in coronavirus and influenza infection [132,133]. Recently, an increase in circulating KP metabolites was shown in COVID-19 patients supporting a role for enhanced AhR activation in response to SARS-CoV-2 [134]. AhR antagonists may thus also provide a novel approach for the treatment of COVID-19 patients [134].

## 6. AhR and Nutritional Therapies

**Figure 3:**
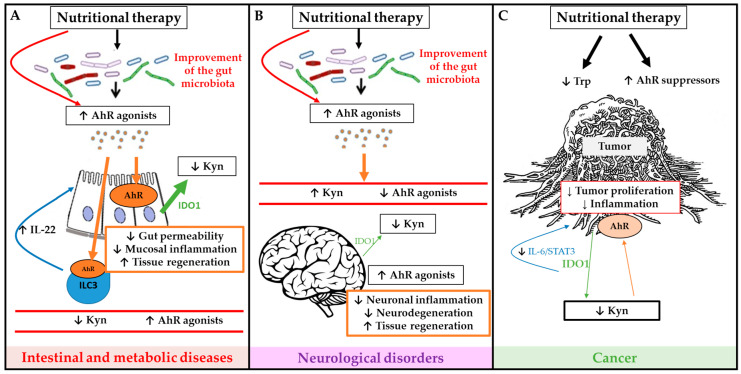
Schematic overview of potential mechanisms of nutritional therapies to modulate microbiota dependent tryptophan (Trp) metabolism and AhR signaling activation in order to improve outcome of intestinal and metabolic diseases (**A**), neurological diseases (**B**) and cancer (**C**). Green arrows represent IDO1 production, large green arrows represent high production and the narrow arrows represent low production. Brown arrows represent AhR activation, large brown arrows represent high activation of AhR, and the narrow arrows represent low activation of AhR.

The main sources of exogenous AhR-ligands are edible plants, vegetables, fruits, teas and herbs [135]. This has raised the hope to develop dietary AhR ligand supplementation to modulate AhR pathway activation in different diseases. Studies in experimental colitis mouse models have shown an attenuation of inflammation after administration of a potent AhR agonist (FICZ), derived from UV light oxidation [72,76]. FICZ also improves glucose metabolism and insulin sensitivity in a metabolic syndrome model [67]. This shows the potential of AhR modulation by supplementation of AhR ligands through the diet. Some of the main in vivo studies in animal models and human diseases are summarized in Table 1. Some of the potential mechanisms of nutritional therapies are summarized in Figure 3.

### 6.1. Tryptophan Supplementation

Different DSS-colitis models have shown positive results upon dietary Trp supplementation. Not only did additional (L-)tryptophan (L-Trp) lead to restoration of AhR ligand production by the gut microbiota, it also increased AhR mRNA expression, decreased the expression of the pro-inflammatory cytokines and upregulated of the anti-inflammatory cytokine IL-22 production in mice and porcine DSS models [136,137]. Additional L-tryptophan in weanling piglets also enhanced intestinal mucosal barrier function by improving tight junctions [138].

In a dietary induced Non-Alcoholic Fatty Liver Disease mice model, TRP supplementation demonstrated a protective effect by stabilizing the intestinal barrier through enhancement of occludin expression and reduction of hepatic fat accumulation [149]. In a mouse model of EAE, supplementation of Trp improved disease scores and limited CNS inflammation in WT mice [102]. However, AhR deficient mice showed no amelioration [102]. This indicates that the effect of Trp was dependent on AhR signaling and that AhR ligand supplementation is also effective for modulating inflammation outside the gut.

Trp metabolism has been shown to be altered in obesity [150]. Studies investigating the effect of intraduodenal L-Trp on metabolism have therefore been done in both lean and (non-diabetic) obese participants. L-trp infusion led to altered gut motor function by suppressing antral and stimulating pyloric pressure, ultimately leading to delayed gastric emptying and subsequently attenuating postprandial blood glucose. L-Trp also affected hormonal function, mainly by regulating cholecystokinin release, and reduced energy intake substantially [151,152].

Glucobrassicin derivatives have also been studied as AhR ligand nutritional supplementation. Glucobrassicin-derived I3C can form into AhR agonists like 2-(indol-3-ylmethyl)−3,3′diindolylmethane, and ICZ, which is considered AhR’s natural ligand with the highest affinity [46]. I3C enrichment has been shown to control intestinal specialized intraepithelial lymphocytes (IEL) development and reduce gut permeability, tissue destruction and macroscopic colitis scores [75,139]. It has also been associated with IL-22 induction, NF-kB suppression, prevention of Th17 expansion, and increased Tregs in the mesenteric lymph node, suggesting that I3C is also effective in directly modulating the immune system [139,153].

In an EAE model, I3C administration led to modulation of clinical symptoms and attenuation of disease by promoting generation of Tregs while downregulating Th17 cells [140]. I3C and its metabolites showed properties to inhibit *Escherichia Coli* and *Staphylococcus biofilm* formation, acting as a strong antimicrobial feature [154].

I3C was also reported to inhibit tumorigenesis in various target tissues [155]. Dietary I3C was associated with suppression of in vivo prostate carcinogenesis by inducing cell cycle arrest and apoptosis of cancer cells [156,157], and also its anti-estrogenic properties leading to inhibition of cell proliferation have been studied in mammary cancer [158]. Importantly, I3C has also been associated with tumorigenesis. Administration of I3C in Rainbow trout fish or rats induced development of colonic lesions and enhanced hepatocarcinogenesis [141,142].

### 6.2. Microbiota-Derived Supplementation

Certain microbiota strains have AhR ligand production capacity and could make good candidates for probiotic administration with the purpose of creating more AhR ligands. *Lactobacillus Reuteri* has been shown to expand upon Trp administration and affect local immune homeostasis [23]. Administration of *Lactobacillus Reuteri* to high fat diet (HFD) fed mice improved intestinal barrier function and secretion of glucagon-like peptide 1, resulting in an improvement of glucose tolerance and liver steatosis. These results were comparable to the effects of FICZ administration in the same group [67]. Oral administration of heat-killed *L. Bulgaricus* shows improvement in DSS-colitis mice by activating the AhR pathway and inducing mRNA expression of *CYP1A1*, one of the AhR pathway target genes [159]. Lamas et al. showed reduced colitis susceptibility in mice by increased *IL22* expression and AhR ligand production upon administration of different *Lactobacillus* strains [40]. In vivo treatments with IAId, one of the Trp derivatives through microbiota modulation, was found to activate AhR and induce IL-22 production [23].

### 6.3. Other AhR Ligands and Remarks

Trp is not the only available naturally occurring ligand. Flavonoids, curcumin, indigoids, retinoids and others have also been studied in the context of their AhR modulating effects [135]. Administration of B-naphtoflavone, an AhR agonist, improved DSS-induced colitis in mice and downregulated colitis-induced pro-inflammatory cytokines such as *TNFα*, *IL6* and *IL1β* mRNA [143]. The identification of increased *AhR* gene expression on tumors and increase of endogenous ligands has led to the research of AhR antagonists, like flavonoids, in the treatment of cancer. Several reports indicate a link between dietary flavonoid intake and potential in cancer treatment [148,160]. However, flavonoids seem to exhibit varying AhR agonist or antagonist activity, depending on the concentration used. Similarly, animal study results show conflicting results, seeming to vary depending on the species used, which makes it difficult to make a recommendation for daily practice [161].

Curcumin has shown promising results in UC. Lang et al. showed that the combination of curcumin with mesalazine was superior to the combination of placebo and mesalamine in inducing clinical and endoscopic remission in patients with mild-to-moderate active UC, yielding no apparent adverse effects [144]. A comprehensive meta-analysis recommended curcumin as a complementary therapy for UC [145]. A recent Japanese randomized placebo-controlled study in patients with active mild-to-moderate Crohn’s disease (CD) showed that the curcumin derivative Theracurmin^®^ (Theravalues, Tokyo, Japan) [162], which has a 27-fold higher absorption rate than natural curcumin powder, improved clinical and endoscopic remission, healing of anal lesions, and blood levels of inflammatory markers [146]. Curcumin also demonstrated great therapeutic potential for metabolic and neurologic diseases. For instance, curcumin supplementation significantly reduced insulin resistance in adults with high risk of type 2 diabetes and Alzheimer’s disease [147].

## 7. Conclusions

Nutritional therapies are taking on a more important role in the treatment of several immune-mediated diseases. In pediatric CD, Exclusive Enteral Nutrition (EEN) and Crohn’s Disease Exclusion Diet (CDED) are used as the first-line treatment [163,164]. The positive results of AhR activation in colitis models and recent clinical studies of curcumin in the management of IBD illustrate the great potential of nutritional modification of AhR signaling.

However, contradicting results regarding potential tumorigenesis of AhR activation require disease- and even cell-type-specific approaches. The linkage of Trp metabolites to a range of diseases has led to substantial efforts to modulate the KP therapeutically, particularly through inhibition of the key enzymes involved, including IDO1 and TDO, although unlike for curcumin, results in clinical trials to date have not been convincing. AhR modulation strategies will increasingly become the focus of translational and therapeutic studies.

## Figures and Tables

**Table 1:** Summary of some of the main nutritional interventions targeting AhR studies in animal models and human diseases.

Species	Models/Diseases	Nutritional Interventions Targeting AhR	Outcomes	Ref
Mice	Dextran sodium sulfate (DSS)-Colitis	L-Trp supplementation	Ameliorated DSS-induced colitis symptoms and severity Increased *AhR* and *IL22* mRNA Decreased pro-inflammatory cytokines	[136]
Piglets	DSS-Colitis	L-Trp supplementation	Ameliorated DSS-induced colitis symptoms and severity Decreased pro-inflammatory cytokines	[137]
Piglets	DSS-Colitis	L-Trp supplementation	Increased abundances of tight-junction proteins	[138]
Mice	Experimental autoimmune encephalomyelitis (EAE)	Trp supplementation	Improved disease scores and limited central nervous system (CNS) inflammation Trp effect was dependent to AhR activation	[102]
Mice	DSS-Colitis	I3C supplementation	Control of intestinal IEL development Reduced gut permeability and tissue destruction I3C effect was dependent to AhR activation Increased Cyp1a1 transcripts	[75]
Mice	2,4,6-Trinitrobenzenesulfonic acid (TNBS)-Colitis	I3C supplementation	Repressed colonic inflammation Prevention of microbial dysbiosis Suppression of Th17 and induction of Tregs Increased IL-22	[139]
Mice	EAE	I3C supplementation	Less clinical symptoms and cellular infiltration into the CNS Suppression of Th17 and induction of Tregs in AhR dependent manner	[140]
Rainbow trout	Aflatoxin B1 (AFB1)-induced hepatocarcinogenesis	I3C supplementation	Aggravated Induced hepatocarcinogenesis AhR signaling was suggested to be involved	[141]
Rat	AOM-induced colon cancer	I3C supplementation	No clear protective or enhancing effect of I3C	[142]
Mice	DSS-Colitis	B-naphtoflavone supplementation	Suppressed DSS-induced colitis Decreased pro-inflammatory cytokines B-naphtoflavone effect was dependent to AhR signaling	[143]
Human	Ulcerative colitis	Curcumin capsules	Enhanced the Mesalamine-induced clinical and endoscopic remission	[144]
Human	Meta-analysis inflammatory bowel diseases (IBD)	Curcumin	Induced remission	[145]
Human	Crohn’s disease	Curcumin derivative Theracurmin^®^	Improved clinical and endoscopic remission Reduced inflammatory markers in blood	[146]
Human	Type 2 diabetes	Curcumin supplementation	Reduced insulin resistance	[147]
Human	Colorectal Cancer	Fisetin (flavonoid) supplementation	Reduced inflammation Fisetin as a complementary antitumor agent	[148]
Mice	Intraperitoneal (i.p.) injection of H22 hepatocellular carcinoma cells i.p. injection of ID8 ovarian cancer cells	i.p. injection of Kyn	Kyn-AhR pathway regulates PD-1 expression in tumor-infiltrating CD8+ T cells	[125]
